# Use of Online or Paper Surveys by Australian Women: Longitudinal Study of Users, Devices, and Cohort Retention

**DOI:** 10.2196/10672

**Published:** 2019-03-14

**Authors:** David Fitzgerald, Richard Hockey, Mark Jones, Gita Mishra, Michael Waller, Annette Dobson

**Affiliations:** 1 Centre for Longitudinal and Life Course Research School of Public Health University of Queensland Brisbane Australia

**Keywords:** online survey, paper survey, longitudinal study, participant retention

## Abstract

**Background:**

There is increasing use of online surveys to improve data quality and timeliness and reduce costs. While there have been numerous cross-sectional studies comparing responses to online or paper surveys, there is little research from a longitudinal perspective.

**Objective:**

In the context of the well-established Australian Longitudinal Study on Women’s Health, we examined the patterns of responses to online or paper surveys across the first two waves of the study in which both modes were offered. We compared the following: differences between women born between 1946 and 1951 and between 1973 and 1978; types of device used for online completion; sociodemographic, behavioral, and health characteristics of women who responded online or using mailed paper surveys; and associations between mode of completion in the first survey and participation and mode of completion in the second survey.

**Methods:**

Participants in this study, who had responded to regular mailed surveys since 1996, were offered a choice of completing surveys using paper questionnaires or Web-based electronic questionnaires starting in 2012. Two groups of women were involved: an older cohort born between 1946 and 1951 aged in their 60s and a younger cohort born between 1973 and 1978 aged in their 30s when the online surveys were first introduced. We compared women who responded online on both occasions, women who responded online at the first survey and used the paper version of the second survey, women who changed from paper to online, and those who used paper for both surveys.

**Results:**

Of the 9663 women in their 60s who responded to one or both surveys, more than 50% preferred paper surveys (5290/9663, 54.74%, on the first survey and 5373/8621, 62.32%, on the second survey). If they chose the online version, most used computers. In contrast, of the 8628 women in their 30s, 56.04% (4835/8628) chose the online version at the first survey. While most favored computers to phones or tablets, many did try these alternatives on the subsequent survey. Many women who completed the survey online the first time preferred the paper version on the subsequent survey. In fact, for women in their 60s, the number who went from online to paper (1151/3851, 29.89%) exceeded the number who went from paper to online (734/5290, 13.88%). The online option was more likely to be chosen by better educated and healthier women. In both cohorts, women who completed paper surveys were more likely than online completers to become nonrespondents on the next survey. Due to the large sample size, almost all differences were statistically significant, with *P*<.001.

**Conclusions:**

Despite the cost-saving advantages of online compared to paper surveys, paper surveys are likely to appeal to a different population of potential respondents with different sociodemographic, behavioral, and health characteristics and greater likelihood of attrition from the study. Not offering a paper version is therefore likely to induce bias in the distribution of responses unless weighting for respondent characteristics (relative to the target population) is employed. Therefore, if mixed mode (paper or online) options are feasible, they are highly likely to produce more representative results than if only the less costly online option is offered.

## Introduction

There is increasing use of online surveys for research to increase participation; improve data completeness, quality, and availability; and reduce costs [1-3]. There have been randomized trials of paper-based surveys compared with online surveys [4-8], nonrandomized studies [9-11], comparisons of different devices for online completion [12], and mixed-methods approaches in which participants are offered both modes simultaneously or sequentially [1,5,9-12].

Researchers usually find that younger and more educated participants favor internet-based modes of completion [13-15], although many participants prefer postal surveys completed with paper and pen [7,14]. Most of the studies have been cross-sectional, and much less has been reported about associations between mode of survey delivery and device use, participant characteristics, and cohort retention in the context of longitudinal studies.

While a mixed mode of data collection has advantages, allowing participants to switch the mode of questionnaire completion has the potential to change the overall and partial response rates, alter representativeness of the sample being assessed, and change the way in which participants respond to particular questions [12,16]. Data from the United Kingdom Household Longitudinal Study (UKHLS) have been used to assess the effect of switching from a face-to-face mode of collection to include telephone or Web-based methods. In some cases, the mixed modes of data collection had a detrimental effect on response rates [16,17]. It has also been hypothesized that the mode of data collection may contribute to differences in the ability of participants to recall events accurately, and the different reliabilities that result from a mixed mode approach may hamper the longitudinal comparisons within a study [18]. To date, results from the UKHLS have shown broad agreement in the accuracy [19] and reliability [18] of responses collected using different modes; however, less research has focused directly on the effects of changing from paper-based surveys to a mixed mode choice of paper and Web-based surveys in a longitudinal study setting [12,20].

By 2012-2013, most households in Australia had internet access, ranging from 85% in the capital cities to 79% in rural areas [21]. Therefore, in order to reduce costs and improve data quality, participants in the Australian Longitudinal Study on Women’s Health, who had responded to regular surveys since 1996, were offered a choice of completing surveys using mailed questionnaires or Web-based electronic questionnaires starting in 2012. Two groups of women were involved: an older cohort born between 1946 and 1951 and a younger cohort born between 1973 and 1978. For this report, our objectives were to examine longitudinal patterns of paper and online responses over the first two waves in which both options were offered:

- Between women born between 1946 and 1951 and between 1973 and 1978
- By type of device used for online completion
- By the sociodemographic, behavioral, and health characteristics of women who responded online or using mailed paper surveys
- Between mode of completion of the first survey and participation and mode of completion in the second survey
- By survey performance criteria, including response rates, noncompletion (break off), missing item responses, and measurement equivalence.

## Methods

### Australian Longitudinal Study on Women’s Health

In 1996, women in three age cohorts—born between 1921 and 1926, born between 1946 and 1951, and born between 1973 and 1978—were recruited to the Australian Longitudinal Study on Women’s Health, with more than 12,000 participants in each cohort responding to the baseline surveys. The sampling frame was the database of the universal national health insurance scheme, now called Medicare, which covers all citizens and permanent residents. Women living in rural and remote areas were recruited at twice the population proportion to ensure sufficient power to detect important differences in health and use of services. Details of the study recruitment, national representativeness, attrition, and measures have been published elsewhere [22,23]. This paper uses data from the women born between 1946 and 1951 and between 1973 and 1978.

### Survey Design

Since the baseline survey in 1996, the women have been resurveyed approximately every three years. Initially paper questionnaires with reply-paid envelopes were mailed to participants. The surveys have many common items but are tailored to address health and health service issues relevant to each cohort and time.

From survey 6 in 2012, women in the 1973-1978 cohort were offered the option of completing the questionnaires online using versions designed for use on mobile phones, tablets, and computers. From survey 7 in 2013, those in the 1946-1951 cohort had the same options. Since then both cohorts have completed another survey enabling us to look at changes in mode of completion and associations between mode of completion and response to the subsequent survey.

At each survey, women who have not previously withdrawn from the study or died and for whom we have current contact details are invited to participate. Since 2012, women for whom we have email addresses have been emailed an invitation and a link to the online survey. If they have not responded after 4 to 6 weeks, during which they are sent email, postal, and text-message reminders, they are mailed paper surveys (followed by email, text-message, and telephone reminders to nonresponders). Women for whom we do not have current email addresses are initially mailed an invitation and the link to the online survey (followed by a mailed or text-message reminder). If they have not completed the online survey within 4 to 6 weeks, they are mailed paper surveys (and followed up using the same reminder protocol as the other group).

When the online surveys were introduced, the paper versions were reformatted to improve questionnaire equivalence. For example, long lists of items that had fit on a page but might not fit on a single screen were broken into shorter lists, each with the same instructions. In addition, several steps were taken to adapt the online surveys to mobile devices. Maximum pixel width for a survey question was limited. Lists of question items with numerous response options that took up a large width were transformed into single items with reduced width that used more scrolling on a mobile device screen. The size of question response controls (radio buttons, checkboxes, buttons, etc) were increased for more accurate touch screen interaction.

### Measurements

The device used by the participant to complete the survey online was determined from the text of the browser’s user agent string. The user agent records which browser and operating system are being used. This information was provided by the online platform used for the surveys. If the user agent string contained the term iPhone, iPad, or Android, the device was categorized as tablet/phone. If the terms Windows, Macintosh, or Linux appeared, the device was categorized as a computer. The device type was considered missing if the user agent string did not contain any of the identified words.

Area of residence was based on a measure of distance from where the woman lived to population centers with varying levels of services (for health, education, shops, etc) [24]. For this paper we used the categories major city, inner regional area, outer regional area, and remote or very remote area. The highest level of education the woman had completed was categorized as school certificate or less, higher school certificate, trade certificate or diploma, or university degree. Marital status was categorized as partnered (married/de facto relationship) or not partnered (separated, divorced, widowed, or never married). A woman’s work pattern was categorized according to her responses to questions about time use. Full-time work was defined as working a total of 35 or more hours per week, and part time work was between 1 and 35 hours; women were classified as not working if they used the response option “I don’t do this activity” to questions about time spent working.

Based on questions about cigarette use, women were categorized as never smoker, former smoker, or current smoker. Alcohol consumption was calculated from questions about frequency and quantity of alcohol consumed and classified according to national guidelines as nondrinker, low-risk drinker or rarely drinks, or risky drinker [25]. At each survey, women reported their height and weight, and these data were used to calculate body mass index (BMI) (kg/m^2^). Each survey included the 36-Item Short Form Health Survey (SF-36) [26]. The item “In general, would you say your health is” with response options excellent, very good, good, fair, or poor was used as a categorical measure of general health. In addition, the physical function and mental health subscales from the SF-36 were calculated.

### Statistical Analysis

To compare modes of completion using the two surveys, matched cross-tabulations and tests for marginal homogeneity (Stuart-Maxwell test) and symmetry were used. To examine the characteristics of women who maintained the same mode of completion versus those who changed mode, four groups were defined: online both times, online followed by paper, paper followed by online, and paper both times. To assess the effect of changing mode of completion on trend estimates, we calculated changes in responses by the same individuals between the two successive surveys before the introduction of the online option and then between the survey immediately before and the survey at which the online option was introduced. Differences between the four groups of women were compared using chi-square tests for categorical variables and one-way analysis of variance (ANOVA) and Tukey tests for continuous variables to identify which groups were statistically significantly different.

## Results

A total of 9663 women in the 1946-1951 cohort responded to surveys 7 or 8 or both. We were unable to identify the device used by 26 of those who completed the survey online at survey 7; for the remaining 9637, the modes of completion and nonresponse are shown in Table 1. Most women used paper surveys on both occasions, with computers the second most frequent method. The use of tablets or phones increased from survey 7 to survey 8, but this remained the less favored mode of completion. While 13.88% (734/5290) of women who used paper at survey 7 changed to an online mode (computer, tablet, or phone) at survey 8, 29.89% (1151/3851) of those who completed survey 7 online chose to use the paper version at survey 8. All these differences were highly statistically significant (*P*<.001). The overall response rates (for all modes of completion) for surveys 6, 7, and 8 were 82.99% (10,011/12,063), 81.05% (8622/10,719), and 80.44% (8622/10,719), respectively. While break off percentages at survey 7 were low (14/3575, 0.39%) for online participants who also responded to survey 8, the percentage was higher (14/286, 4.90%) for women who did not respond at survey 8. Similarly, the percentage of women with missing responses for more than 10% of items at survey 7 was low (116/8109, 1.43%) among respondents in all four mode of completion groups who also responded to survey 8, but it was higher for those who did not respond to survey 8 (6.6% [19/286] for those who responded online to survey 7 and 6.5% [49/756] for those who responded on paper).

**Table 1:** Comparison of mode of completion of survey 7 in 2013 and survey 8 in 2016 women in the 1946-1951 cohort of the Australian Longitudinal Study on Women’s Health.

	Survey 8				
Survey 7	Computer	Tablet/phone	Paper	Nonresponse	Total
Computer	1772 (51.80)	369 (10.79)	1022 (29.87)	258 (7.54)	3421 (100)
Tablet/phone	79 (19.08)	184 (44.44)	123 (29.71)	28 (6.76)	414 (100)
Paper	533 (10.08)	201 (3.80)	3800 (71.83)	756 (14.29)	5290 (100)
Nonresponse	60 (12.72)	24 (4.69)	428 (83.59)	—	512 (100)
Total	2444 (25.36)	778 (8.07)	5373 (55.75)	1042 (10.81)	9637 (100)

Characteristics of women in the 1946-1951 cohort in the four main groups are compared in Table 2. While women who responded online in one or both surveys were very similar, women who used the paper survey both times were more likely to live in regional (but not remote) areas, less educated, less likely to be working, more likely to be smokers, and less likely to drink alcohol. These women were also more likely to be overweight or obese and had poorer health on all measures. Like the women who changed from paper to online completion, the paper-only group were less likely to have partners than the groups of women who completed survey 7 online. All differences were statistically significant at *P*<.001.

Table 3 shows that these differences between the groups were (retrospectively) evident even at the baseline survey in 1996. The Tukey tests showed that the differences were consistently greater between the women in the paper-only group and the other three groups. They were slightly older (even though all women were within a 5-year age range), had higher BMIs, and had poorer levels of physical function and mental health. In contrast, the changes over time were of similar magnitude in all four groups. That is, although the levels of these variables were different at baseline, they tracked approximately in parallel over time. While the changes between surveys 5 and 6 appear somewhat different from the changes between surveys 6 and 7, possibly due to the mode change, the effects were similar across all four groups of women (except for a greater decline in physical function in the paper-only group).

Among the women born between 1973 and 1978, 8628 completed one or both of surveys 6 and 7. For 10 of the online responders we could not identify the device they used, but for the remainder the modes of completion and nonresponse are shown in Table 4. As expected, this younger group of women were more likely than the 1946-1951 cohort to complete the surveys online and to use tablets or phones. Also, unlike the older group, more changed from paper to online surveys (1006/3164, 31.80%) than went from online to paper (963/4835, 19.92%, of those who completed survey 6 online).

**Table 2:** Comparison of women in the 1946-1951 cohort of the Australian Longitudinal Study on Women’s Health who responded to survey 7 in 2013 and survey 8 in 2016 (N=8109), according to their modes of survey completion.

Characteristics		Online/online (n=2424^a,b^), n (%)	Online/paper (n=1151^c,b^), n (%)	Paper/online (n=734^b^), n (%)	Paper/paper (n=3800^b^), n (%)
**Area of residence**					
	Major city	1055 (44.03)	456 (39.93)	317 (43.37)	1314 (34.84)
	Inner regional	898 (37.48)	461 (40.37)	283 (38.71)	1572 (41.69)
	Outer regional	396 (16.53)	195 (17.08)	118 (16.14)	794 (21.06)
	Remote/very remote	47 (1.96)	30 (2.63)	13 (1.78)	91 (2.41)
**Educational qualifications**					
	School certificate or less	572 (25.05)	336 (31.26)	193 (28.34)	1620 (47.07)
	Higher school certificate	426 (18.66)	208 (19.35)	121 (17.77)	695 (20.19)
	Trade certificate/diploma	545 (23.87)	265 (24.65)	182 (26.73)	664 (19.29)
	University degree	740 (32.41)	266 (24.74)	185 (27.17)	463 (13.45)
**Marital status**					
	Partnered	1825 (75.63)	925 (81.07)	534 (73.25)	2774 (73.48)
	Not partnered	588 (24.37)	216 (18.93)	195 (26.75)	1001 (26.52)
**Work**					
	Full-time	370 (15.33)	200 (17.53)	143 (19.51)	504 (13.37)
	Part-time	791 (32.77)	354 (31.03)	248 (33.83)	1045 (27.72)
	Not working	1253 (51.91)	587 (51.45)	342 (46.66)	2221 (58.91)
**Smoking**					
	Never smoked	1496 (61.95)	728 (63.80)	494 (67.49)	2401 (63.60)
	Former smoker	824 (34.12)	372 (32.60)	213 (29.10)	1053 (27.89)
	Current smoker	95 (3.93)	41 (3.59)	25 (3.42)	321 (8.50)
**Alcohol consumption**					
	Nondrinker	244 (10.10)	123 (10.76)	90 (12.31)	797 (21.32)
	Low-risk/rarely drinks	1997 (82.66)	962 (84.16)	600 (82.08)	2732 (73.09)
	Risky drinker	175 (7.24)	58 (5.07)	41 (5.61)	209 (5.59)
**General health**					
	Excellent/very good	1441 (59.50)	619 (53.87)	363 (49.52)	1512 (39.89)
	Good	782 (32.29)	415 (36.12)	297 (40.52)	1659 (43.77)
	Fair/poor	199 (8.22)	115 (10.01)	73 (9.96)	619 (16.33)

^a^There were 20 women who responded online to both surveys who were not included in Table 1 because it was not possible to know if they had used a computer, tablet, or phone in survey 7.

^b^Not all women responded to every item.

^c^There were 6 women who responded online for survey 7 and used paper for survey 8 who were not included in Table 1 because it was not possible to know if they had used a computer, tablet, or phone on survey 7.

**Table 3:** Comparison of measures at surveys 1 (1996), 6 (2010), 7 (2013), and 8 (2016) from women in the 1946-1951 cohort of the Australian Longitudinal Study on Women’s Health who responded to surveys 7 and 8, according to their mode of survey completion.

Characteristics		Online/online (n=2424)	Online/paper (n=1151)	Paper/online (n=73)	Paper/paper (n=3800)	*P* value
Age at survey 1, mean		47.44	47.45	47.45	47.68	<.001
**Body mass index**						
	Survey 1	25.40	25.45	25.51	25.92	<.001
	Change from survey 6 to survey 7	0.09	0.23	0.23	0.17	.20
	Change from survey 7 to survey 8	–0.01	0.01	–0.01	0.09	.23
**SF-36^a^** **subscale for physical function**						
	Survey 1	88.94	88.00	88.91	86.40	<.001
	Change from survey 6 to survey 7	–1.75	–2.07	–1.07	–1.65	.61
	Change from survey 7 to survey 8	–1.07	–1.97	–1.40	–2.34	.02
**SF-36 subscale for mental health**						
	Survey 1	75.89	75.09	75.52	74.24	<.001
	Change from survey 6 to survey 7	0.72	1.00	0.99	0.56	.77
	Change from survey 7 to survey 8	–0.39	–0.68	–0.18	0.07	.35

^a^SF-36: 36-Item Short Form Health Survey.

**Table 4:** Comparison of modes of completion of survey 6 in 2012 and survey 7 in 2015 women in the 1973-1978 cohort of the Australian Longitudinal Study on Women’s Health.

	Survey 7				
Survey 6	Computer	Tablet/Phone	Paper	Nonresponse	Total
Computer	1981 (46.52)	833 (19.56)	846 (19.87)	598 (14.04)	4258 (100)
Tablet/Phone	137 (23.74)	229 (39.69)	117 (20.28)	94 (16.29)	577 (100)
Paper	595 (18.81)	411 (12.99)	1408 (44.50)	750 (23.70)	3164 (100)
Nonresponse	203 (32.79)	126 (20.36)	290 (46.85)	—	619 (100)
Total	2916 (33.84)	1599 (18.55)	2661 (30.88)	1442 (16.73)	8618 (100)

The overall response rates (for all modes of completion) for surveys 5, 6, and 7 were 62.01% (8200/13,223), 61.63% (8010/12,997), and 56.61% (7186/12,693), respectively, of all eligible women. While break off percentages at survey 6 were low (36/4152, 0.87%) for online participants who also responded to survey 7, the percentage was higher (43/692, 6.21%) for women who did not respond at survey 7. Similarly, the percentage of women with missing responses for more than 10% of items at survey 7 was low (91/6567, 1.39%) among respondents in all four mode of completion groups who also responded to survey 8, but it was higher for those who did not respond to survey 7 (7.4% [51/692] for those who responded online at survey 6 and 3.2% [24/750] for those who responded on paper).

The women born between 1973 and 1978, who were aged 34 to 39 years when they responded to survey 6 in 2012, were more urban, much better educated, more likely to work full-time or part-time, and generally in better health (see Table 5) than the women in the 1946-1951 cohort, who were aged 62 to 67 years when they responded to their survey 7 in 2013. The younger women were more homogeneous than the older women, but differences between the four response groups followed broadly the same patterns. Most of the differences were still highly statistically significant (*P*<.001), although the differences were less pronounced for some characteristics (eg, for marital status, *P*=.006) and the differences between the groups on general health were not statistically significant (*P*=.15).

As for the older cohort, these differences between the groups were (retrospectively) evident even at the baseline survey in 1996 (see Table 6). The differences were mainly that the women in the online-only group had better physical function and mental health than women in the other three groups. While the changes over time differed between surveys 5 and 6 and surveys 6 and 7 (suggestive of a possible mode of completion effect), they were of similar magnitude in all four groups of women.

**Table 5:** Comparison of women in the 1973-1978 cohort of the Australian Longitudinal Study on Women’s Health who responded to survey 6 in 2012 and survey 7 in 2015 (N=6567), according to their modes of survey completion.

Characteristics		Online/online (n=3189^a,b^), n (%)	Online/paper (n=963^b^), n (%)	Paper/online (n=1007^c,b^), n (%)	Paper/paper (n=1408^b^), n (%)
**Area of residence**					
	Major city	1917 (64.68)	539 (57.16)	543 (54.96)	749 (53.92)
	Inner regional	701 (23.65)	261 (27.68)	272 (27.53)	397 (28.58)
	Outer regional	289 (9.75)	132 (14.00)	147 (14.88)	217 (15.62)
	Remote/very remote	57 (1.92)	11 (1.17)	26 (2.63)	26 (1.87)
**Educational qualifications**					
	School certificate or less	108 (3.41)	44 (4.64)	50 (5.04)	127 (9.20)
	Higher school certificate	297 (9.39)	110 (11.59)	118 (11.88)	200 (14.49)
	Trade certificate/diploma	786 (24.84)	257 (27.08)	263 (26.49)	418 (30.29)
	University degree	1973 (62.36)	538 (56.69)	562 (56.60)	635 (46.01)
**Marital status**					
	Partnered	2493 (78.79)	785 (82.72)	808 (80.24)	1078 (76.95)
	Not partnered	671 (21.21)	164 (17.28)	199 (19.76)	323 (23.05)
**Work**					
	Full-time	1429 (45.15)	368 (38.78)	389 (38.71)	493 (35.09)
	Part-time	1189 (37.57)	414 (43.62)	432 (42.99)	626 (44.56)
	Not working	547 (17.28)	167 (17.60)	184 (18.31)	286 (20.36)
**Smoking**					
	Never smoked	2008 (63.32)	601 (62.93)	635 (63.18)	855 (60.85)
	Former smoker	875 (27.59)	272 (28.48)	265 (26.37)	353 (25.12)
	Current smoker	288 (9.08)	82 (8.59)	105 (10.45)	197 (14.02)
**Alcohol consumption**					
	Nondrinker	330 (10.41)	102 (10.68)	115 (11.44)	209 (14.98)
	Low-risk/rarely drinks	2697 (85.08)	804 (84.19)	859 (85.47)	1127 (80.79)
	Risky drinker	143 (4.51)	49 (5.13)	31 (3.08)	59 (4.23)
**General health**					
	Excellent/very good	1979 (62.21)	600 (62.50)	593 (58.95)	818 (58.18)
	Good	943 (29.64)	284 (29.58)	323 (32.11)	465 (33.07)
	Fair/poor	259 (8.14)	76 (7.92)	90 (8.95)	123 (8.75)

^a^There were 9 women who responded online to both surveys who were not included in Table 5 because it was not possible to know if they had used a computer, tablet, or phone on survey 7.

^b^Not all women responded to survey.

^c^There was 1 woman who used paper on survey 6 and responded online on survey 7 who was not included in Table 5 because it was not possible to know if she had used a computer, tablet, or phone in survey 7.

**Table 6:** Comparison of measures at surveys 1 (1996), 5 (2009), 6 (2012) and 7 (2015) from women in the 1973-1978 cohort of the Australian Longitudinal Study on Women’s Health who responded to surveys 6 and 7, according to their mode of survey completion.

Characteristics		Online/online (n=3189)	Online/paper (n=963)	Paper/online (n=1007)	Paper/paper (n=1408)	*P* value
**Age at survey 1, mean**		20.75	20.79	20.81	20.87	.08
**Body mass index**						
	Survey 1	22.81	22.24	22.73	22.77	.003
	Change from survey 5 to survey 6	0.36	0.44	0.42	0.50	.37
	Change from survey 6 to survey 7	0.60	0.60	0.61	0.55	.92
**SF-36^a^** **subscale for physical function**						
	Survey 1	92.61	92.45	91.67	91.40	<.001
	Change from survey 5 to survey 6	0.14	0.25	–0.22	0.57	.73
	Change from survey 6 to survey 7	–0.45	–0.14	0.58	–0.28	.35
**SF-36 subscale for mental health**						
	Survey 1	70.65	69.93	68.13	68.22	.008
	Change from survey 5 to survey 6	0.03	0.14	0.25	0.53	.82
	Change from survey 6 to survey 7	–1.07	–1.06	–1.70	–1.68	.50

^a^SF-36: 36-Item Short Form Health Survey.

## Discussion

### Principal Findings

Introducing the choice of online or paper completion of successive surveys in a well-established longitudinal study enabled us to examine how participants would respond over time. As expected, we found that the online option was more likely to be chosen by younger, better educated women. Surprisingly, we found that a large number of women who completed the survey online the first time this was offered preferred the paper version on the subsequent survey. In fact, for women in their 60s, the number who went from online to paper exceeded the number who went from paper to online.

Overall the women in their 60s tended to prefer to continue with paper surveys, and if they chose the online version most used computers. In contrast, the younger women, in their 30s, were more likely to adopt the online version and, while most favored computers to phones or tablets, many did try these alternatives at the subsequent survey.

In this study, it is not uncommon for participants to miss one (or more) of the triennial surveys but complete subsequent surveys. We found that women who completed paper surveys were more likely than online completers to become nonrespondents at the next survey.

Although there was some evidence of an effect of introducing the online option on the temporal changes in key variables, the effects were similar for women who made different choices about mode of completion. That is, there was little evidence of confounding between self-selection of mode of completion and measurement in these health-related variables. Changing the format of the paper surveys to be more like the format of the online surveys may have achieved measurement equivalence in the same survey wave but introduced discontinuity over time.

### Limitations

In this paper, we report survey response behavior of women in their 30s and 60s in Australia. The results may not be generalizable to men, people in other age groups, or people with less Web access. Nevertheless, this is a large study with more than 18,000 participants who were originally randomly selected from almost all women in Australia in their age groups at baseline in 1996. Over time, national migration and study attrition have changed the representativeness of the study population, favoring more educated and Australian-born women, but the resultant bias is unlikely to affect the direction of the main results [27].

Although internet access in Australia was more than 80% when the online surveys were first introduced, it may not have been sufficiently reliable for completion of a long survey. At the end of each survey (paper or online), women are encouraged to provide additional comments and suggestions in free text format. While many women do provide comments on a variety of topics, fewer than five mentioned the mode of completion, mainly reporting they had experienced difficulties accessing the online version.

### Comparison With Prior Work

Most previous studies comparing online and paper survey respondents have been cross-sectional. They have found similar results to ours: younger and more educated people are more likely to respond online [13-15]. This effect was strong and consistent in our data for women born between 1946 and 1951 but less pronounced in the younger, more homogeneous cohort. Preference for paper questionnaires and postal surveys has also been found by others [7,13,14]. What our study adds is the longitudinal aspect, showing that respondents may choose to swap between online and paper modes of completion at successive surveys. While this swapping behavior might be due in part to the time when participants received the invitations, it also suggests that many women preferred the paper format, which may enable slower, more deliberative response.

As others have reported [12], we found that online and paper responses produced equivalent scores on key health-related variables reported at the same time. While formatting the paper survey to more closely mimic the online version may have enhanced measurement equivalence across different groups of women, it may also have biased estimates of change over time.

### Conclusions

Despite the cost-saving advantages of online compared to paper surveys, we found the latter appeal to a different population of potential respondents. This difference is likely to induce bias in the distribution of responses unless weighting for respondent characteristics (relative to the target population) is employed. From a longitudinal perspective, we also found that women who completed paper surveys were less likely to respond to the subsequent survey, adding to the potential bias. In conclusion, if mixed mode options are feasible, they are highly likely to produce more representative results than if the only response option is online.

## Abbreviations

ANOVA: analysis of variance
BMI: body mass index
SF-36: 36-Item Short Form Health Survey
UKHLS: United Kingdom Household Longitudinal Study
